# Supplementary material for: Alterations in Progesterone Receptor Isoform Balance in Normal and Neoplastic Breast Cells Modulates the Stem Cell Population
Source: Cells. 2020 Sep 11;9(9):2074. doi: 10.3390/cells9092074 (PMC7564437; doi:10.3390/cells9092074)
Supplement: Supplementary file 1 [file cells-09-02074-s001.pdf]

Supplementary Figures

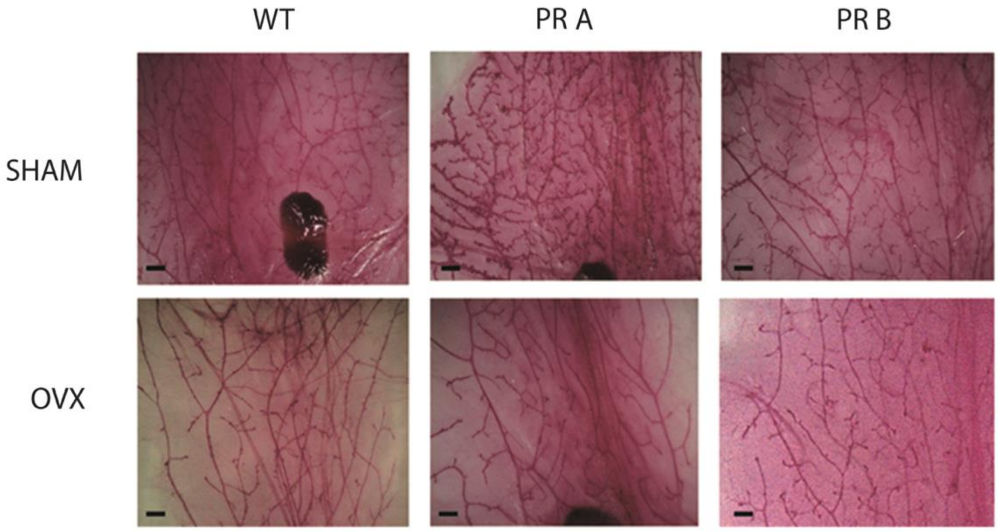

Figure S1. Impact of ovariectomy on mammary glands of WT, PRA, and PRB mice. Representative mammary gland whole mounts of sham operated and ovariectomized (OVX) 20-week-old WT, PRA, and PRB transgenic mice. Scale bar: 500 µm.

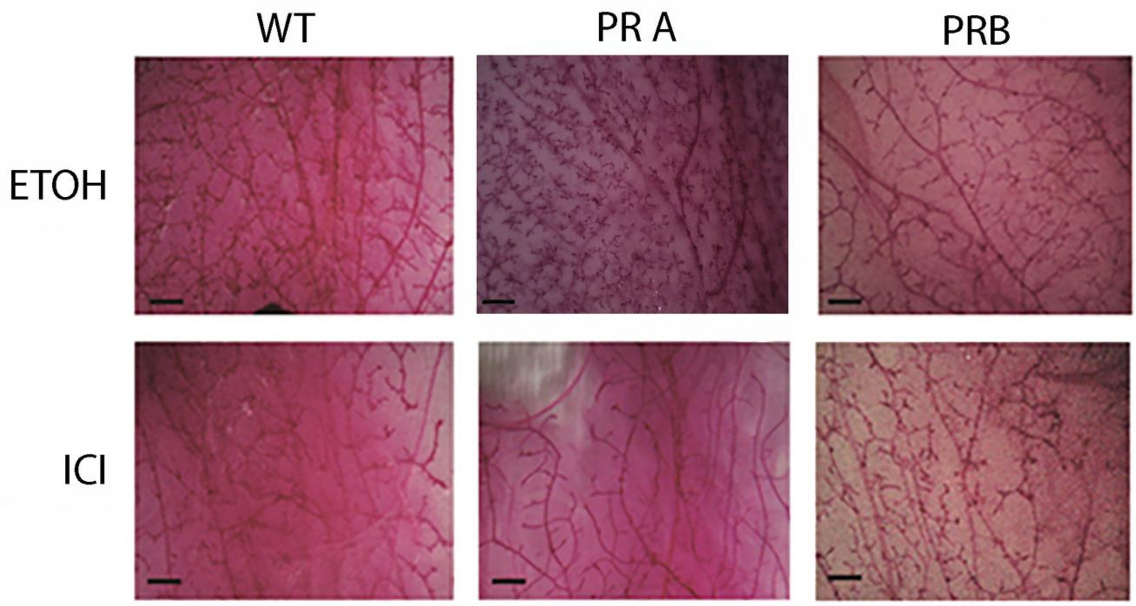

Figure S2. Impact of ICI 180, 780 treatments on mammary glands of WT, PRA, and PRB transgenic mice. Representative mammary gland whole mounts of vehicle (ETOH) and ICI 180, 780 (ICI) treated 20-week-old WT, PRA, and PRB transgenic mice. Scale bar: 500 µm.

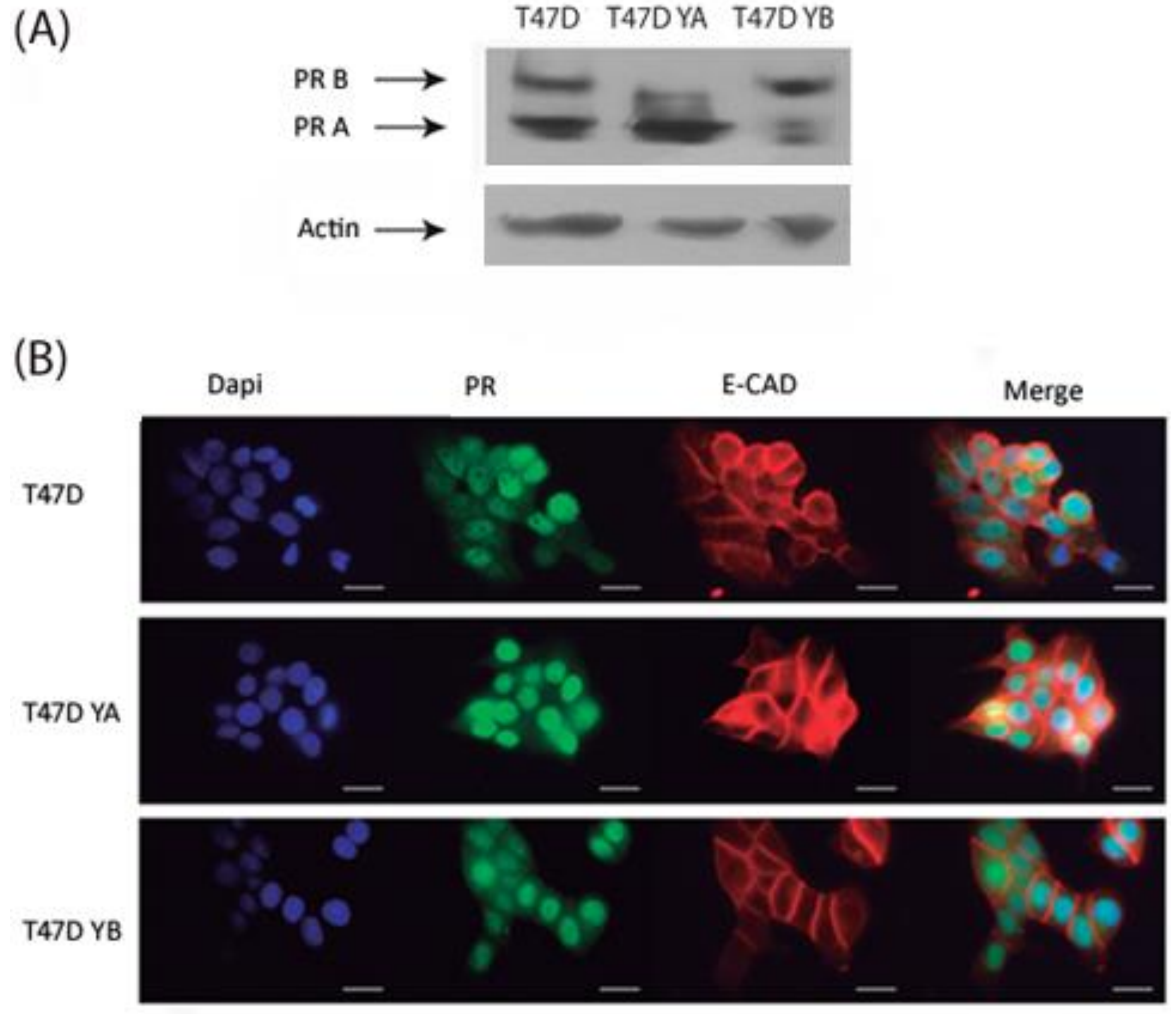

Figure S3. T47D model. (**A**) Western Blot of protein extracts derived from T47D, T47D YA and YB cell lines for PRA and B and ER-α. Actin was used as loading control. (**B**) Immunofluorescence against PR and E-Cadherin. Nuclei were stained with DAPI. Scale bar: 50 μm.
